# Effects of Yeast Culture on Lamb Growth Performance, Rumen Microbiota, and Metabolites

**DOI:** 10.3390/ani15050738

**Published:** 2025-03-05

**Authors:** Jinlong Xu, Xiongxiong Li, Qingshan Fan, Shengguo Zhao, Ting Jiao

**Affiliations:** 1 College of Pratacultural Science, Gansu Agricultural University, Lanzhou 730070, China; xjl_gsau@163.com (J.X.); fanqsh18@lzu.edu.cn (Q.F.); 2 Key Laboratory for Grassland Ecosystem of Ministry of Education, Gansu Agricultural University, Lanzhou 730070, China; 3 Provincial R&D Institute of Ruminants in Gansu, Lanzhou 730070, China; zhaosg@gsau.edu.cn; 4 College of Animal Science and Technology, Gansu Agricultural University, Lanzhou 730070, China; lxx_gsau@163.com

**Keywords:** lamb, yeast culture, growth performance, rumen tissue, rumen microorganisms, microbial metabolism

## Abstract

The effects of incorporating yeast culture (YC) into pelleted feeds on sheep production and the potential impact on rumen microbial populations, microbial metabolism, and fermentation have not been extensively studied. This study aimed to evaluate the effect of YC on growth performance, rumen tissue development, rumen fermentation, and rumen microflora in sheep and to explore the potential microbial mechanisms involved. Fifty healthy 3-month-old male lambs of small-tailed Han sheep, with an average weight of 28.44 ± 0.63 kg, were randomly divided into five groups: control (0% YC), 3% YC, 6% YC, 9% YC, and 12% YC. The pre-feeding period lasted for 15 days, followed by an official feeding period of 60 days. On the last day of the formal feeding period, six lambs that exhibited the best growth performance were randomly selected from the control group and the 9% YC group. These sheep were slaughtered, then the rumen epithelial tissue and rumen contents were collected for the measurement of rumen fermentation, microbial populations, and metabolites. Compared to the control group, the YC-treated groups showed higher daily and final body weight gains, as well as increased levels of propionic acid, butyric acid, and total volatile fatty acids (*p* < 0.05). YC supplementation also enhanced rumen papilla length and width (*p* < 0.05). Additionally, YC increased the relative abundance of certain microbial species (*p* < 0.05). These results suggest that supplementing 9% YC in pelleted diets for small-tailed Han sheep may enhance growth performance and improve the rumen environment.

## Simple Summary

Modern feeding methods and high-grain diets have negative effects on the rumen health of ruminants. Yeast culture (YC) is a kind of green additive probiotic rich in yeast polysaccharides, mannan oligosaccharides, vitamins, peptides, and organic acids. These components can be used as fermentation substrates and growth factors to promote the growth of beneficial bacteria in the gastrointestinal tract of ruminants to effectively improve their gastrointestinal health. Therefore, this study aimed to scientifically verify the theoretical basis for the effect of YC in maintaining the rumen of lambs and promote partial replacement of antibiotics for lamb fattening and health maintenance in practice. The lambs were divided into five groups, fed 0%, 3%, 6%, 9%, and 12% YC. The basic diet was the same, and the test period was 70 days. By measuring the production performance, health indicators, and rumen environment of lambs, it was found that the 9% YC changed the rumen fermentation environment of lambs, promoted the development of rumen tissue, improved feed utilization, and ultimately improved the fattening effect. The results suggest that adding 9% YC to pelleted diets at high concentrations helps promote the development of rumen epithelium and regulates the fermentation pattern of the rumen, thereby maintaining rumen health in sheep.

## 1. Introduction

The rumen is a unique digestive organ in ruminants, playing a critical role in the digestion of fiber and the absorption of nutrients by the host. This organ houses a complex and diverse microbiota that interacts synergistically to form a highly efficient “natural fermenter”, capable of breaking down cellulose in food [1]. The health and function of the rumen not only directly impact the physiological status of ruminants but are also closely linked to their production performance [2]. Therefore, it is essential to maintain the rumen’s internal environment. However, modern intensive feeding practices and the widespread use of high-grain diets have negatively affected sheep rumen health [3]. The use of antibiotics in animal husbandry to deal with the production problems of sheep promotes the development of antibiotic-resistant strains, and imbalance in the rumen microbiome may also pose a risk to human health [4]. In recent years, the application of plant-derived natural substances and probiotics have gained recognition as effective strategies to replace antibiotics in maintaining ruminant health [5,6]. Among these, yeast culture (YC), a green additive probiotic, is rich in yeast polysaccharides, mannan oligosaccharides, vitamins, peptides, and organic acids. These components can serve as fermentation substrates and growth factors, promoting the growth of beneficial bacteria in the gastrointestinal tract of ruminants, thereby effectively improving their gastrointestinal health [7,8].

Years of research have shown that YC has a positive impact on the health and growth of ruminants [9,10]. It can promote the development of the rumen epithelium of male fattening sheep, improve nitrogen utilization, and improve the rumen microbial environment by increasing the relative abundance of *Succiniclasticum* and *Fibrobacter* [11]. Furthermore, Pi et al. [12] found that adding YC can stabilize the pH value of the rumen by reducing lactic acid-producing bacteria and increasing lactic acid-utilizing bacteria or rumen protozoa, thereby determining the rumen environment. Further results from Li et al. [13] indicated that the addition of YC improved fermentation parameters by modulating the Anaplasma phage, *Prevotella_*7, and *Lachnospiraceae_*NK3A20 groups in lamb rumen, thereby enhancing the flavor of Aohu sheep. Supplementation with yeast culture (YC) also enhanced the abundance of fibrinolytic microbiota and enriched metabolites in rumen associated with amino acid and lipid metabolism pathways of male Hu lambs, thereby maintaining rumen homeostasis under high-concentrate dietary conditions [14]. Liu et al. [15] found that the concentrations of acetic acid and total volatile fatty acids (TVFAs) in the rumen of small-tailed Han sheep increased, the concentration of pH decreased, and the relative abundance of *Lachnospiraceae_*NK3A20 and *Olsenella* increased when using a high-concentration diet. The polysaccharide components in yeast cultures modulate the structure and metabolism of the rumen microbiota, thereby increasing nutrient utilization in ruminants and improving the quality of livestock-derived products [16]. Although existing studies have demonstrated the benefits of yeast culture (YC) in regulating rumen microbial communities, nitrogen utilization efficiency, and volatile fatty acid profiles across various ruminant species, the feasibility of incorporating YC into pelleted diets and its mechanistic impacts on energy metabolism pathways through functional gene–metabolite interactions remain less explored. This limits the widespread adoption of YC as a green natural additive with properties of health maintenance and regulation of the rumen environment, integrated into pellet feed, in intensive lamb production systems.

This study hypothesized that supplementing pelleted feed with brewer’s yeast culture (YC) might improve growth performance and maintain rumen health by altering rumen bacteria and metabolites, which could positively affect rumen tissue morphology in mature sheep. Therefore, this study aimed to evaluate the impact of YC supplementation in pelleted feed on growth performance, rumen tissue development, rumen fermentation indices, rumen bacterial composition, and metabolomics in small-tailed Han sheep.

## 2. Materials and Methods

### 2.1. Yeast Culture

Yeast culture (YC) is produced using distiller grains as the fermentation substrate, which is inoculated with high-activity probiotics such as *Bacillus subtilis*, *Saccharomyces cerevisiae*, and *Aspergillus cerevisiae*. The production process involves using composite strains inoculated with liquid culture, followed by secondary solid fermentation for 48 to 72 h. The YC was provided by Sichuan Runge Biotechnology Co., Ltd., Mianzhu City, China. The number of viable bacteria reached 1 × 10^8^ CFU/g, and the inoculation rate of composite strains was 10%. The nutritional active ingredients of YC include crude protein (dry matter basis) ≥ 17%, crude fiber ≤ 20%, crude ash ≤ 12%, crude fat ≥ 2.6%, and total organic acid ≥ 5%.

The study was conducted at Yingqian Agricultural and Animal Husbandry Development Co., Ltd. in Linxia City, China. This experiment has been approved by the Animal Ethics Committee of Gansu Agricultural University (GSAU-2nd-AST-2023-053). Fifty healthy 3-month-old male small-tailed Han lambs (average weight: 28.44 ± 0.63 kg) were randomly divided into five groups: control (CON) and 3%, 6%, 9%, and 12% YC. Each treatment group (YC) consisted of 10 sheep, which were housed in separate single pens with one replicate per sheep.

The trial utilized a nutritionally balanced total mixed ration (TMR) formulated in compliance (PRC NY/T 816-2004), with detailed nutritional composition presented in Table 1. Animals received TMR rations twice daily (08:00 and 18:00) ad libitum, with graded supplementation of *Saccharomyces cerevisiae* culture (YC; viable count ≥ 1 × 10^8^ CFU/g) at 3%, 6%, 9%, and 12% (dry matter basis). The 15-day adaptation phase was followed by a 60-day test period. At the end of the test, the 9% YC group exhibited the greatest daily weight gain, the fastest growth rate, and the greatest final weight. Consequently, six lambs were randomly selected from both the 9% YC experimental and control groups for slaughter and further analysis.

### 2.2. Sample Collection and Processing

Rumen fluid and epithelial tissues were collected from the rumen after lambs were slaughtered. A total of 70 mL of rumen content was obtained from each lamb and filtered through four layers of gauze. The collected rumen fluid was then divided into three portions: one portion was transferred to a 10 mL cryotube for DNA extraction, another portion was allocated to a separate 10 mL cryotube for metabolite identification, and the final portion was placed into a 50 mL centrifuge tube for the assessment of rumen fermentation parameters. Once dispensed, the samples were transported to the laboratory in liquid nitrogen tanks and stored in an ultralow-temperature freezer at −80 °C for future analysis.

### 2.3. Product Performance

Throughout the treatment phase, both the initial individual body weight and the final body weight were recorded. A daily log was maintained to track the amount of feed consumed, from which the average daily feed intake (ADFI), average daily gain (ADG), and feed-to-gain ratio (F/G) were calculated.

### 2.4. Determination of Rumen Histomorphometry

Five tissue samples, each measuring 2.5 × 2.5 cm, were excised from the abdominal capsule region of each sheep. The rumen and abdominal tissues were immersed in 4% paraformaldehyde for 24 h, after which paraffin sections were prepared by orienting the specimens in a water-based medium within a ventilated environment. The process for sectioning comprised sampling, dehydration, waxing, embedding, and sectioning, all conducted in a ventilated setting. After hematoxylin–eosin (HE) staining, the sections were dehydrated and sealed with a mixture of anhydrous ethanol and xylene. The rumen papilla’s length and width and the muscle layer’s thickness were then measured using Case Viewer software (Case Viewer 2.4.0), followed by image acquisition using a Panoramic 250 digital slide scanner (3DHISTECH, Budapest, Hungary).

### 2.5. Rumen Fermentation Characteristics

Rumen pH was determined using a calibrated digital pH meter (P611, Shanghai Leici Instrument Co., Ltd., Shanghai, China) equipped with a temperature-compensated electrode, with triplicate measurements recorded within 2 min post-sampling to minimize atmospheric exposure. The concentration of ammonia nitrogen (NH₃-N) in the rumen was quantified using established colorimetric methods, performed with the assistance of a 721-type spectrophotometer (TU-1901). Additionally, volatile fatty acids (VFAs) were analyzed using gas chromatography with a GC-2010 Plus from Shimadzu (Kyoto, Japan). These methodologies ensure accurate and reliable assessments of the chemical composition of the rumen fluid [19].

### 2.6. High-Throughput Sequencing and Analysis

Microbial DNA was extracted from rumen content samples using an MN NucleoSpin 96 Soil kit (Macherey-Nagel, Düren, Germany), and the concentration and purity were assessed using a NanoPhotometer (N60, Düren, Germany). The community structure of the rumen microbiota was determined by PCR amplification of the V3–V4 region of the 16S rRNA gene. The primers used were 338F (5′-ACTCCTACGGGAGGCA GCAG-3′) and 806R (5′-GGACTACHVGGGTWTCTAAT-3′). The library obtained from PCR amplification was sequenced on an Illumina MiSeq platform (Illumina, San Diego, CA, USA), and bioinformatic analysis was performed using BMKCloud.

The raw data produced by the Illumina MiSeq platform underwent a series of processes, including the merging of paired-end reads (using FLASH v1.2.7), filtering (via Trimmomatic v0.33), and removal of chimeric sequences (with UCHIME v4.2) to derive optimized sequences, known as tags. Subsequently, Usearch software (v11 64bit) [20] was employed to cluster these tags at a similarity threshold of 97%, generate operational taxonomic units (OTUs), and taxonomic annotation of the OTUs was performed using the Silva (bacteria) taxonomic database. The OTU assessment results were analyzed at various taxonomic levels, resulting in community structure maps for each sample by phylum, class, order, family, genus, and species. Alpha diversity metrics were applied to evaluate species diversity within individual samples, yielding indices such as Ace, Chao1, Shannon, and Simpson. Sample dilution curves [21] and rank abundance curves were also constructed. Beta diversity analyses were carried out to investigate variations in species diversity, particularly in the composition and structure of the microbiota. A PCA map was generated based on the distance matrix [22], and biomarkers with statistically significant differences between groups were identified through intergroup significance analysis (LEfSe analysis) [23]. Metastats software (QIIME2 2023.2) was used for t-tests on species abundance data across groups to derive *p*-values [24], which were subsequently adjusted to yield q-values. Ultimately, species responsible for compositional differences between the two sample groups were identified based on the q-values. Functional analysis of the 16S gene was conducted using the Kyoto Encyclopedia of Genes and Genomes (KEGG) framework and the Clusters of Orthologous Groups (COGs).

### 2.7. Metabolomic Analysis of Rumen Fluid

Rumen fluid specimens were passively thawed at 4 °C following extraction from a −80 °C cryostorage system. Aliquots (500 μL) were homogenized with an equivalent volume of acetonitrile–methanol (1:1, *v*/*v*) solvent mixture and vortex-mixed for 30 sec, followed by 10 min sonication (PS-60AL apparatus) and 60 min incubation at −20 °C. Particulate removal was achieved through dual-phase centrifugation (13,000 rpm, 4 °C, 15 min). Chromatographic separation was performed on a Shimadzu LC-30 platform equipped with an Acquity UPLC HSS T3 column (2.1 × 100 mm, 1.8 μm), employing the aqueous mobile phase at a 0.3 mL/min flow rate with column thermostating at 50 °C, utilizing 2 μL injection volumes. High-resolution mass spectrometric detection was implemented using a Triple TOF 5600 system operated in dual ionization modes. Raw spectral data processing through MassLynx V4.2 incorporated peak annotation and spectral alignment, with metabolite identification via Progenesis QI cross-referenced against the METLIN and Biomark proprietary databases, maintaining stringent mass accuracy thresholds (<100 ppm). Pathway mapping and compound classification were executed through KEGG, HMDB, and Lipid MAPS resources, with differential analysis performed using two-tailed T-tests to determine statistical significance (*p*-value computation for all analytes).

### 2.8. Statistical Analysis

The statistical analyses were conducted utilizing SPSS v.27.0 (IBM, Armonk, NY, USA) and R v.4.0.2 (R Foundation, Vienna, Austria). This investigation systematically assessed multiple variables encompassing forage nutritional composition, rumen fermentation characteristics, microbial community structure (analyzed at phylum and genus taxonomic levels), fibrinolytic enzyme kinetics, CAZyme gene expression profiles, and rumen histomorphometric parameters. Comparative analyses between experimental groups were performed using Student’s t-test, with statistical significance defined at α = 0.05 (two-tailed). Nonparametric Wilcoxon rank-sum tests were implemented for pathway enrichment analysis of differentially expressed genes in metabolic pathways. Bivariate associations between microbial taxa (top 20 operational taxonomic units) and differentially abundant metabolites were quantified using Spearman’s rank correlation analysis (ρ coefficients ranging −1 to +1). Data visualization was executed using ggplot2 and related packages within the R programming environment. This multiparametric analytical framework facilitated a comprehensive evaluation of interrelationships among nutritional, microbial, enzymatic, and histological parameters in the rumen ecosystem.

## 3. Results

### 3.1. Growth Performance Analysis

As shown in Table 2, the final body weight and average daily gain in the 9% YC group were significantly higher than those of the control group (*p* < 0.05). At the same time, the average daily weight gain was the highest in the 9% YC group, with the lowest feed-to-gain ratio.

### 3.2. Differences in VFAs in Sheep Rumen

The concentrations of TVFA, propionic acid, and butyric acid were significantly higher in the 9% YC group than those of the control group, while the ethyl–propyl ratio was considerably lower in the 9% YC group compared to the control group (Table 3; *p* < 0.05).

### 3.3. Differences in Rumen Organization

**Figure 1:**
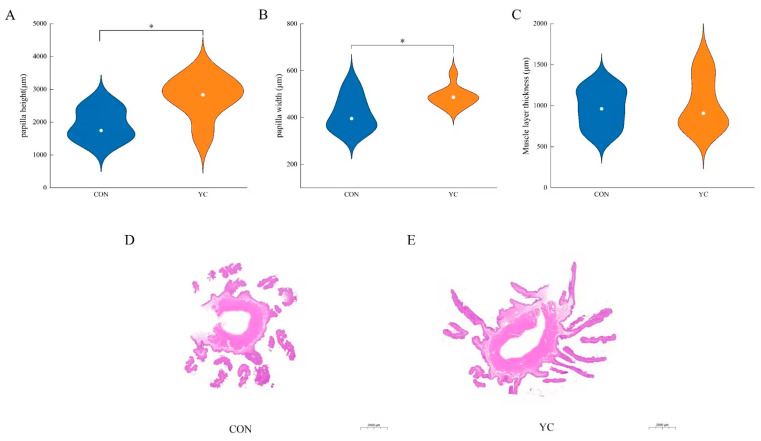
(**A**–**C**) Measurement of indices related to rumen morphology. (**D**,**E**) H&E staining of the ruminal wall. CON = control group; YC = yeast culture group. * Significant difference between groups (*p* < 0.05).

The papilla height and width in the rumen of sheep in the 9% YC group were significantly higher than those in the control group (Figure 1 and Table S1).

### 3.4. Analysis of the Alpha Diversity of Rumen Microorganisms

**Figure 2:**
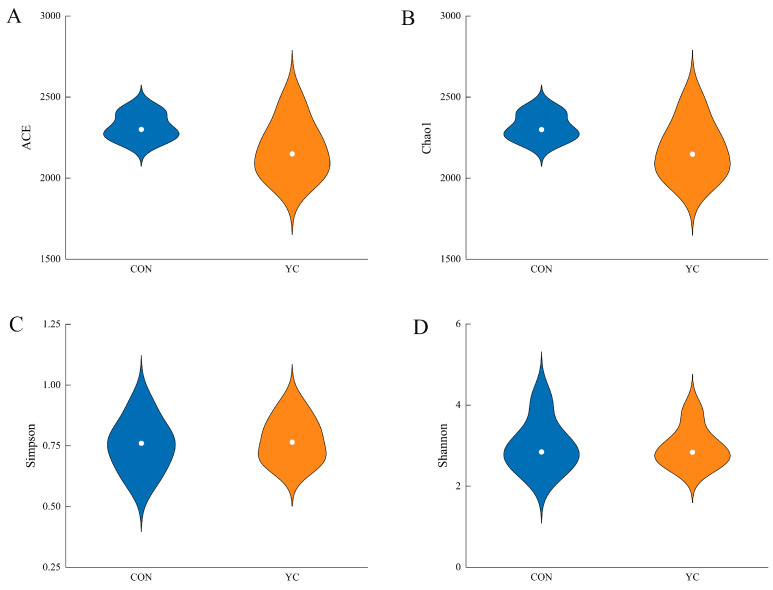
Alpha diversity of rumen microorganisms in both groups. (**A**), ACE index. (**B**), Chao 1 index. (**C**), Simpson index. (**D**), Shannon index. CON = control group; YC = yeast culture group.

There was no significant difference between the two groups in rumen microbiota alpha diversity analysis, including the Shannon, Simpson, ACE, and Chao1 indices (*p* > 0.05) (Figure 2).

### 3.5. Differences in the Composition of Rumen Microbial Communities in Sheep

**Figure 3:**
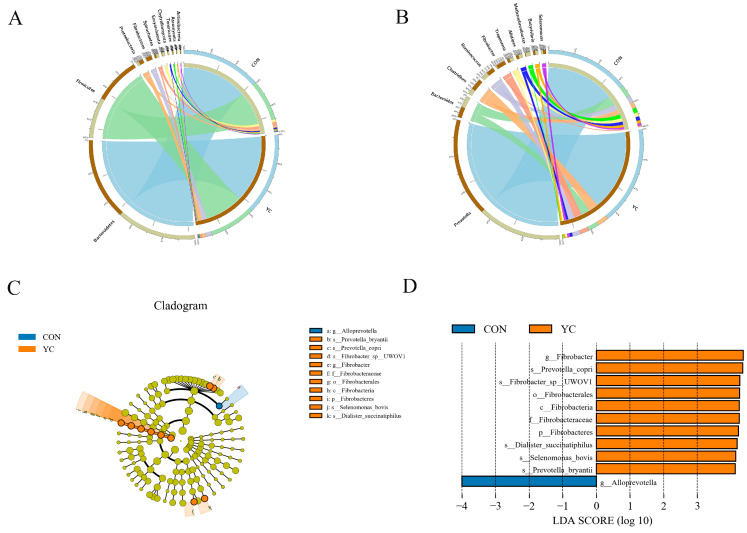
Relative abundance of phylum (**A**) and genus (**B**) level species. (**C**) LEfSe analysis of branching diagrams. (**D**) Significantly different bacterial taxa were identified by linear discriminant analysis effect size (LEfSe) (threshold value set to 3.0). CON = control group; YC = yeast culture group.

At the phylum level, rumen bacteria mainly included Bacteroidetes, Firmicutes, Proteobacteria, and Fibrobacteres (Figure 3A). The relative abundance of Fibrobacteres in the 9% YC group was significantly higher than that in the control group (Table S2, *p* < 0.05).

At the genus level, rumen bacteria primarily consist of *Prevotella*, *Bacteroides*, *Clostridium*, *Ruminococcus*, and *Fibrobacter* (Figure 3B). The relative abundance of *Clostridium*, *Chlamydia*, and *Fibrobacter* was significantly higher in the 9% YC group than that in the control group (Supplementary Table S3, *p* < 0.05). Conversely, *Alloprevotella*, *Candidatus_Nanosyncoccus*, and *Tannerella* were significantly higher in the control group than those in the YC group (Table S3, *p* < 0.05).

LEfSe difference analysis was performed to examine the rumen microorganisms identified in the two groups (Figure 3C). The results of the LEfSe analysis (Figure 3D) revealed that the genera with higher abundance in the control group included Alloprevotella. In contrast, the genera with higher abundance in the YC group were Fibrobacteres, Dialister_succinatiphilus, Selenomonas_bovis, Prevotella_bryantii, and Prevotella_copri.

### 3.6. Differences in CAZy and KEGG Pathways of Sheep Rumen

The concentrations of glycoside hydrolases (GHs), glycosyltransferases (GTs), polysaccharide lyases (PLs), and carbohydrate esterases (CEs) were higher in the rumen of sheep in the YC group than in the control group (*p* > 0.05) (Supplementary Table S3). The GH, GT, PL, and CBM families associated with polysaccharide degradation were compared. The relative abundance of CBM50, GH23, GH73, GH24, GT9, GT83, GT32, GH4, GH84, CBM41, CBM88, CBM26, GT49, GH108, GH100, and GH108 was significantly higher in the rumen of sheep in the YC group compared to the control group. Conversely, the relative abundance of GH171, CBM12, CBM66, CBM77, and PL4 in the rumen of control sheep was considerably higher than that in the YC group (Figure S1).

**Figure 4:**
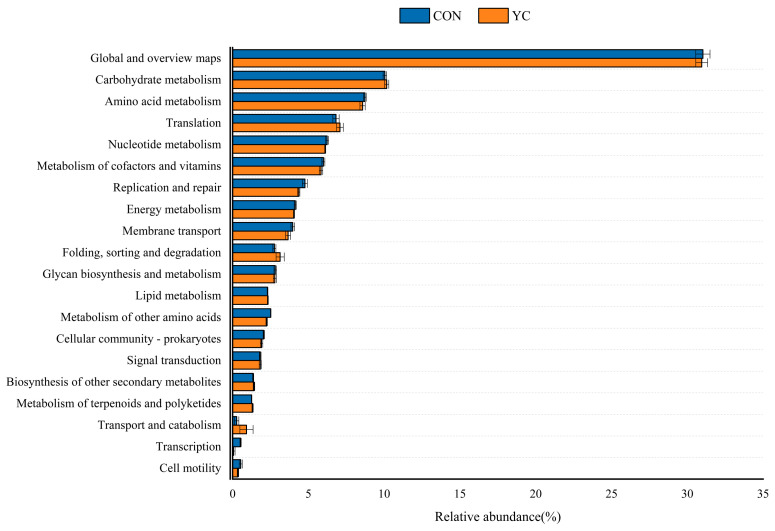
Functional genes related to the KEGG secondary metabolic pathway in both groups. CON = control group; YC = yeast culture group.

At level 2 (Figure 4), the global and overview maps showed pathways including carbohydrate metabolism, amino acid metabolism, translation, nucleotide metabolism, metabolism of cofactors and vitamins, replication and repair, energy metabolism, membrane transport, folding, sorting and degradation, glycan biosynthesis and metabolism, lipid metabolism, metabolism of other amino acids, cellular community—prokaryotes, signal transduction, biosynthesis of other secondary metabolites, metabolism of terpenoids and polyketides, transport and catabolism, transcription, and cell motility (*p* > 0.05).

### 3.7. Differences in Rumen Metabolites

**Figure 5:**
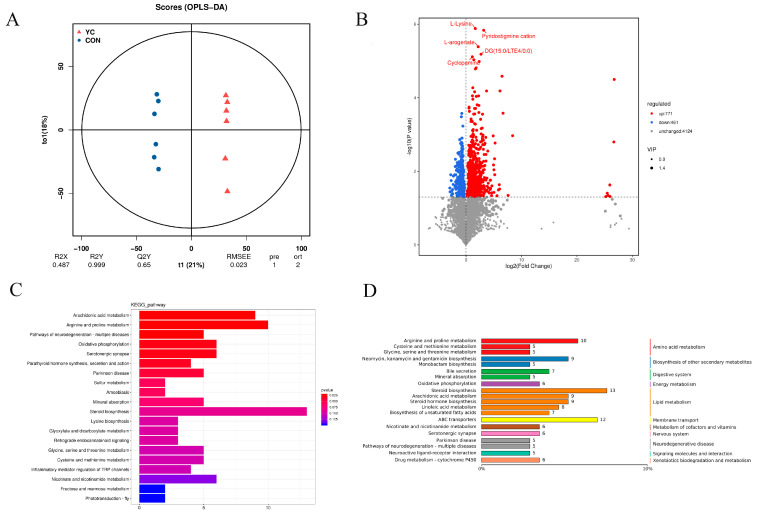
(**A**) OPLS-DA model score plots in the two groups. (**B**) Volcano maps of differential metabolites in the two groups. (**C**) KEGG map of enriched pathways. (**D**) Classification diagram of differential metabolites. CON = control group; YC = yeast culture group.

In the OPLS-DA model, the R^2^Y value for comparison approached 1 between the two groups, while the slope of the fitted regression line for Q^2^Y was positive. This indicated that the established model was both stable and reliable, making it suitable for comparing the differences between the two groups (Figure 5A). Using a significance threshold of *p* < 0.05, we identified 1232 differential metabolites between the two groups, of which 771 were significantly upregulated and 461 were significantly downregulated (Figure 5B).

KEGG enrichment analysis was conducted to elucidate the metabolic pathways associated with the significantly different metabolites. The key differential metabolites predominantly pertained to amino acid metabolism, digestion, lipid metabolism, and energy metabolism pathways (Figure 5D). Notably, the primary differential metabolic pathways identified between the two groups included arachidonic acid metabolism, arginine and proline metabolism, neurodegenerative pathways related to multiple diseases, serotonergic synapses, and oxidative phosphorylation (Figure 5C).

### 3.8. Correlation Analysis

**Figure 6:**
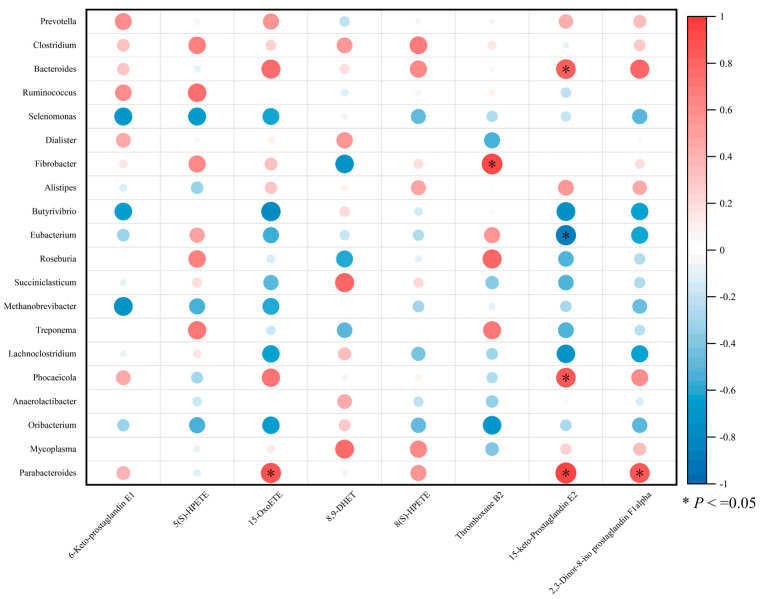
Microbiome–metabolome correlation heatmap.

Fibrobacter was significantly and positively correlated with thromboxane B2 (*p* < 0.05). Parabacteroides were significantly and positively correlated with 15-OxoETE and 2,3-dinor-8-iso prostaglandin F1 alpha (*p* < 0.05). Additionally, 15-keto-prostaglandin E2 was positively correlated with Bacteroides, Phocaeicola, and Parabacteroides, but negatively correlated with Eubacterium (*p* < 0.05) (Figure 6).

## 4. Discussion

The YC used in this study reached ≥1 × 10⁸ CFU/g and produced abundant cellular metabolites, including organic acids, oligosaccharides, and vitamins. During the experiment, the addition of 9% YC to the pelleted feed significantly enhanced the sheep’s daily gain and final body weight. However, it is noteworthy that feed intake did not result in significant differences between the two experimental groups, suggesting that the improvement in growth performance in ruminants by adding YC to pelleted feeds is not dependent on changes in feed intake. This finding is consistent with the results reported by Qi and Chen et al. [12,25]. We hypothesize that this effect may be closely related to the role of YC in regulating rumen fermentation, rumen microbial communities, and their metabolites.

Ruminants degrade cellulose, hemicellulose, and carbohydrates in their feed through rumen microbes to produce volatile fatty acids (VFAs), which are essential for energy and growth [26]. Previous studies have shown that adding yeast cultures to lamb rations improves rumen fermentation performance and VFA absorption, while also enhancing the rumen fermentation environment and promoting rumen development [14,27]. In this study, we found that supplementing yeast cultures in pelleted feeds significantly increased the concentrations of propionic acid, butyric acid, and total short-chain fatty acids (SCFAs), while also decreasing the acetic acid-to-propionic acid ratio. The entry of yeast fermentation metabolites into the sheep rumen significantly increased the abundance of fibrinolytic bacteria and improved the breakdown and utilization efficiency of neutral and acidic detergent fibers. Yeast cultivation promotes the growth of lactic acid-utilizing bacteria by altering the structure of the rumen microbiota, thereby utilizing the lactic acid produced in the rumen to increase rumen pH and promote rumen fermentation while maintaining a stable rumen environment [15]. Most of the VFAs produced by fermentation are absorbed through the rumen epithelium and play a key role in regulating the energy supply for the host’s life activities [28]. The addition of 9% yeast culture to pelleted feed significantly increased butyric and propionic acid content, which in turn stimulated rumen tissue development, significantly increasing the height and width of rumen papillae [29,30]. This suggests that yeast culture supplementation may enhance the efficient absorption, utilization, and transport of VFAs in sheep.

Macro-genomic sequencing was employed to gain deeper insights into the impact of adding yeast cultures to pelleted feed on the profile and regulation of rumen bacteria in sheep. The findings revealed that the predominant bacterial populations at the phylum level in the rumen of both experimental groups were primarily from Bacteroidetes and Firmicutes. The yeast culture contains a cellulase system, hemicellulase (xylanase), and neutral protease. Hemicellulase and cellulase have higher activity, so they can help improve the digestion of the cellulose components in the diet [14]. In this study, the relative abundance of Fibrobacter in the 9% YC group significantly increased, producing various cellulases and hemicellulases that helped to break down cellulose and hemicellulose in plant cell walls. These can break down plant cell walls into fermentable sugars (such as glucose), thereby increasing the content of acetic acid in the rumen fermentation parameter of the 9% YC group [31,32,33]. *Clostridium* generates substantial short-chain fatty acids (SCFAs), particularly butyric acid, through carbohydrate fermentation [34]. This observation explains the marked increase in butyric acid levels following yeast culture supplementation, which subsequently promotes mitotic activity in rumen wall tissues [35]. The enhanced relative abundance of *Prevotella* in the 9% YC group led to increased propionate production. The liver uses propionic acid as a raw material for gluconeogenesis, providing more energy to the body, resulting in higher daily weight gain in the 9% YC group compared to other groups [36,37]. Meanwhile, *Prevotella* utilizes nitrogen sources such as ammonia nitrogen and amino acids to convert non-protein nitrogen in the rumen into high-quality microbial protein that can be utilized by animals, thereby improving the utilization rate of feed nitrogen [1]. This may indicate the effective utilization of nitrogen in microbial protein synthesis and reduction of nitrogen loss and also explains the level of histidine nitrogen in the 9% YC group being lower than that in the control group.

The influence of yeast cultures on the abundance of rumen bacteria may be attributed to the presence of mannan oligosaccharides and glucans in the yeast cell wall. These components are readily degraded in the rumen and serve as sources of fermentable metabolites, minerals, and enzymes, significantly altering the characteristics and physiological functions of rumen bacteria. This alteration can lead to increased proliferation of bacteria specializing in the hydrolysis of cellulose, starch, and protein, thereby enhancing the overall efficiency of nutrient utilization during digestion [10,38]. Yeast cultures play a vital role in fostering the growth of lactic acid-utilizing bacteria, essential for maintaining a stable rumen pH. This stabilization creates an optimal environment for the development of bacteria involved in cellulose breakdown, as well as those that produce acetic and propionic acids. Previous research has shown that YC significantly increases the population of cellulolytic bacteria, lactate-utilizing bacteria, and enzymes involved in carbohydrate metabolism in the rumen. Additionally, another study indicated that the metabolites present in yeast cultures can promote the growth of cellulolytic bacteria [39,40]. The findings from these studies align with our research, reinforcing the beneficial effects of yeast cultures on rumen microbiota. In an intriguing development, our study demonstrated that the incorporation of yeast cultures led to a decrease in the relative abundance of Pseudomonas *Methanobrevibacter*, which consequently resulted in lower methane production in the rumen of sheep. This reduction not only mitigated energy loss for the host but also contributed to a decrease in greenhouse gas emissions. Fibrobacter, comprising core fiber-degrading bacteria in the rumen, efficiently breaks down difficult-to-digest fibers, thereby reducing fiber retention time and subsequently decreasing total methane production [41]. Additionally, Clostridium species utilize the butyrate kinase pathway, which consumes H₂ during butyrate formation, potentially reducing the availability of methane precursors and consequently decreasing methane emissions [42,43]. Previous studies have revealed a strong negative correlation between the number of *Prevotella* species and methane emissions. It has been suggested that members of the *Prevotella* genus can utilize hydrogen to produce propionic acid, thereby reducing methane output [44,45]. Our findings are consistent with these studies, further emphasizing the potential of yeast cultures to enhance rumen efficiency while reducing environmental impacts.

A metabolomic approach based on untargeted LC-MS was used to analyze changes in rumen metabolites. Our study showed that the addition of YC affected rumen metabolites, which mainly consisted of amino acids and their metabolites, as well as lipids and their metabolites. YC modulates rumen metabolite profiles primarily by altering rumen microorganisms, ultimately impacting the host animal [14]. We further analyzed the functional enrichment of differential metabolites between the two groups of sheep. The YC group showed higher enrichment scores in the arachidonic acid and arginine and proline metabolic pathways. Further analysis of the key metabolites in these pathways revealed that the arachidonic acid metabolic pathway in the YC group exhibited higher enrichment scores for metabolites such as 6-keto-prostaglandin E1, 15-OxoETE, 5(S)-HPETE, 8,9-DHET, 8(S)-HPETE, thromboxane B2, 15-keto-prostaglandin E2, and 2,3-dinor-8-iso prostaglandin F1 alpha, which were significantly upregulated. Previous studies have shown that arachidonic acid enhances animal immunity and energy metabolism [33,46,47]. Previous studies have demonstrated that YC supplementation significantly impacts rumen metabolism. In dairy goats, YC primarily influenced pathways related to lipid metabolism, glycan biosynthesis, amino acid metabolism, and vitamin-cofactor metabolism [39]. Similarly, in sheep fed high-concentrate diets, YC supplementation altered the profile of key metabolites, including amino acids, organic acids, carbohydrates, and glycerophosphates [14]. These metabolic changes reflect the fundamental relationship between feed composition and rumen microbial community structure, where microbial cross-feeding interactions determine the overall metabolic capacity of the rumen ecosystem [29]. Our findings align with these previous observations, particularly in the enhanced amino acid and lipid metabolism pathways observed in the 9% YC group. In this experiment, some metabolites belonging to the arachidonic acid signaling pathway were significantly elevated in sheep fed YC-supplemented pelleted diets, suggesting that YC can promote fatty acid and amino acid metabolism through rumen microorganisms and metabolites. This in turn enhances the ability of sheep to utilize arachidonic acid, an essential fatty acid, thereby improving energy utilization and production performance.

## 5. Conclusions

In summary, YC can significantly promote the development of rumen tissues in sheep, improve rumen fermentation and microbial flora structure, decrease the abundance of flora associated with starch degradation, and increase the abundance of flora associated with fiber catabolism. These changes in the microbiota further affected rumen metabolism, leading to differences in metabolites related to amino acid and lipid metabolism. The results suggest that adding 9% YC to pelleted diets at high concentrations helps promote the development of rumen epithelia and regulates the fermentation pattern of the rumen, thereby maintaining rumen health in sheep.

## Figures and Tables

**Table 1:** Basic diet formulation (dry matter basis).

Items	Diets (%)	Nutrient Levels	
Corn	32.0	Digestive energy DE/(MJ/kg)	11.02
Wheat bran	6.0	Moisture content/%	11.76
Grass meal	10.0	CP/%	16.43
Wheatgrass	10.0	Ash/%	8.16
Soybean meal (43%)	5.0	Salinity/%	0.96
Cottonseed meal (46%)	5.0	Ca/%	1.23
Bran	8.0	P/%	0.49
Corn germ meal	15.0	NDF/%	37.84
DDGS (distiller dried grains with solubles)	5.0	ADF%	26.28
Premix ^a^	4.0	NFE (g/kg) ^b^	617.92

^a^ Composition (per kg of dry matter): 100,000–500,000 IU of vitamin A, 50,000–200,000 IU of vitamin D3, ≥500 IU of vitamin E, Fe 1500–7000 mg, Cu 300–750 mg, Mn 1000–5000 mg, Zn 1500–4000 mg, I 20–30 mg, Se 5–20 mg, Co 8–35 mg. ^b^ NFE (g/kg DM) = 1000 − (EE + CP + CF + Ash) [17,18]. CP: crude protein; NDF: neutral detergent fiber; ADF: acid detergent fiber.

**Table 2:** Effect of adding yeast culture on the growth performance of lambs.

Items	CON	3% YC	6% YC	9% YC	12% YC	*p* Value
Initial weight (kg)	27.33 ± 0.58	27.77 ± 0.81	28.23 ± 0.82	28.47 ± 0.72	27.5 ± 0.57	0.764
Final weight (kg)	42.9 ± 0.25 ^b^	44.87 ± 1.11	45 ± 0.89	46.57 ± 0.80 ^a^	45.02 ± 0.39	0.040
ADG (g/d)	277.98 ± 24.13	305.36 ± 27.50	299.40 ± 25.59	323.21 ± 28.19	312.80 ± 29.18	0.232
ADFI (kg/d)	1.61 ± 0.09	1.62 ± 0.07	1.59 ± 0.1	1.67 ± 0.13	1.66 ± 0.09	0.977
F/G	5.79 ± 0.31	5.31 ± 0.24	5.32 ± 0.35	5.17 ± 0.41	5.30 ± 0.28	0.706

Note: CON = control group; YC = yeast culture group. ^a,b^ Values within a row with different letters are significantly different (*p* < 0.05).

**Table 3:** Effect of the addition of yeast culture on rumen fermentation.

Items	CON	9% YC	*p* Value
pH	6.65 ± 0.16	6.44 ± 0.15	0.346
NH_3_-N (mg/dL)	8.70 ± 2.50	5.93 ± 0.55	0.051
TVFA (mmol/L)	67.41 ± 2.30 ^b^	81.91 ± 3.47 ^a^	0.006
Acetate (mmol/L)	41.86 ± 0.73	47.79 ± 3.13	0.119
Propionate (mmol/L)	14.30 ± 1.59 ^b^	19.11 ± 0.99 ^a^	0.002
Butyrate (mmol/L)	8.69 ± 1.13 ^b^	11.91 ± 0.76 ^a^	0.039
Isobutyric acid (mmol/L)	0.73 ± 0.08	0.78 ± 0.05	0.644
Valerate (mmol/L)	0.711 ± 0.02	0.78 ± 0.04	0.151
Isovalerate (mmol/L)	1.12 ± 0.20	1.54 ± 0.14	0.124
A/P	2.96 ± 0.13	2.51 ± 0.12	0.028

Note: CON = control group; YC = yeast culture group. TVFA: total volatile fatty acid. A/P: acetic acid-to-propionic acid ratio. ^a,b^ Values within a row with different letters are significantly different (*p* < 0.05).

## Data Availability

The data presented in this study are available on request from the corresponding author.

## Supplementary Materials

The following supporting information can be downloaded at: https://www.mdpi.com/article/10.3390/ani15050738/s1, Figure S1: Top 10 carbohydrate differences; Table S1: Differences in rumen tissue structure; Table S2: Differences in rumen bacterial phyla (Abundance >1%) and genera (top 20); Table S3: Differences in carbohydrate-active enzymes.
