# Supplementary figures and images for: Effects of Yeast Culture on Lamb Growth Performance, Rumen Microbiota, and Metabolites
Source: Animals (Basel). 2025 Mar 5;15(5):738. doi: 10.3390/ani15050738 (PMC11899153; doi:10.3390/ani15050738)

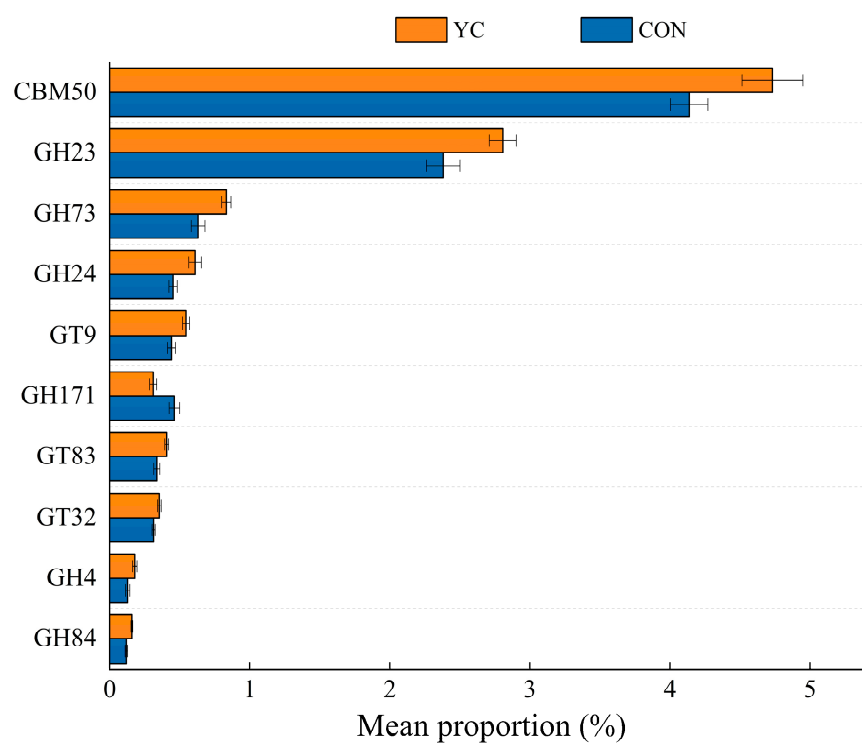

**Supplement Figure 1**, Top 10 carbohydrate differences

Supplement: Supplementary file 1 [file animals-15-00738-s001.zip › supplemental figure.pdf]
